# Arsenic in Drinking Water, Transition Cell Cancer and Chronic Cystitis in Rural Bangladesh

**DOI:** 10.3390/ijerph121113739

**Published:** 2015-10-28

**Authors:** Mohammad Golam Mostafa, Nicola Cherry

**Affiliations:** 1 National Institute of Cancer Research, Dhaka1212, Bangladesh; E-Mail: mohammadgolammostafa@gmail.com; 2 Department of Medicine, University of Alberta, Edmonton, AB T6G 2T4, Canada

**Keywords:** arsenic, drinking water, Bangladesh, chronic cystitis, transition cell cancer, over-matching

## Abstract

In earlier analyses, we demonstrated dose-response relationships between renal and lung cancer and local arsenic concentrations in wells used by Bangladeshi villagers. We used the same case-referent approach to examine the relation of arsenic to biopsy confirmed transition cell cancer (TCC) of the ureter, bladder or urethra in these villagers. As the International Agency for Research on Cancer (IARC) has conclude that arsenic in drinking water causes bladder cancer, we expected to find higher risk with increasing arsenic concentration. We used histology/cytology results from biopsies carried out at a single clinic in Dhaka, Bangladesh from January 2008 to October 2011. We classified these into four groups, TCC (*n* = 1466), other malignancies (*n* = 145), chronic cystitis (CC) (*n* = 844) and other benign (*n* = 194). Arsenic concentration was estimated from British Geological Survey reports. Odds ratios were calculated by multilevel logistic regression adjusted for confounding and allowing for geographic clustering. We found no consistent trend for TCC with increasing arsenic concentration but the likelihood of a patient with benign disease having CC was significantly increased at arsenic concentrations >100 µg/L. We conclude that the expected relationship of TCC to arsenic was masked by over-matching that resulted from the previously unreported relationship between arsenic and CC. We hypothesize that CC may be a precursor of TCC in high arsenic areas.

## 1. Introduction

In a previous publication we assessed the relationship between arsenic in drinking water in villages in Bangladesh and cancers of the kidney, looking at both renal cell cancers (RCC) and transition cell cancers (TCC) [1]. Using a case-referent design, we found a clear dose response, with the risk (odds ratio) for both RCC and TCC increasing with the concentration of arsenic in well water estimated for the “thana” (the smallest administrative area in Bangladesh) of residence. In the current report we have turned our attention to TCCs elsewhere in the urinary tract, particularly in the urinary bladder. This secondary analysis seemed less likely to result in new knowledge: although there were no data published for Bangladesh, the International Agency for Research on Cancer (IARC) had concluded in 2004 that there was sufficient evidence in humans that arsenic in drinking water caused TCC of the urinary bladder and a further evaluation, citing more recent studies, reached the same conclusion in 2009 [2,3]. There have recently been a number of systematic reviews/meta-analyses concerned to establish if there was evidence of effect at concentration below 50 μg/L and to extrapolate evidence from high exposures to predict life-time risk of bladder cancer at the US drinking standard of 10 µg/L [4,5,6]. All such reviews were in concordance with the conclusion that exposure to high concentrations of arsenic increases the risk of bladder (transition cell) cancer.

The three main confounders for studies of urinary tract cancer are age, sex and smoking tobacco [7]. Men are more likely to be diagnosed with urinary tract cancer than women (with an odds ratio of about 2.0) [8]. Bladder cancer incidence increases sharply after the age of 60 years for both men and women [9]. In a meta-analysis of smoking and urinary tract cancer, current smokers had an increased risk of 2.8 (men) and 2.3 (women) with former smokers having a lower but still increased risk (men 2.0; women 1.7) [10]. Over 90% of the cases in the studies included in this review were of the bladder and a sensitivity analysis showed that tumour site did not affect the summary odds ratio for tobacco smoking. It is suggested that there may be a latency of several decades between the onset of smoking and the development of bladder cancer [8]. The incidence rate for bladder cancer is higher in more developed countries [11] with age standardized estimates for Bangladesh in 2012 being only around a quarter of those in the UK [12]. Schistosomiasis, related to bladder cancer in some low income countries, is not endemic in Bangladesh [13].

The current study considers the relation between arsenic concentrations in drinking water and urinary tract cancers in Bangladesh, where contamination of drinking water is widespread. In 1998–1999 a survey commissioned by the People’s Republic of Bangladesh from the British Geological Survey (BGS) found that 27% of hand pumped wells had concentrations of arsenic >50 µg/L [14] (the WHO guideline is 10 µg/L [15]). The data from the 1998–1999 survey have previously been used to examine the relationship between arsenic and lung cancer and renal cancer [1,16]. These studies, using a case-referent design, compared estimates of arsenic in the district or thana of residence for cases (biopsies read as malignant) and referents (biopsies read as benign) reviewed at a single centre serving the whole country. The present study used the same approach to estimate the relationship between arsenic concentration in well water and TCC in the urinary tract other than the kidney.

## 2. Methods

The Anowara Diagnostic Clinic, established by one of us (MGM) in 2002, has kept detailed records of all patients. It provides a service of histopathology and cytology that is poorly accessible elsewhere in Bangladesh: in 2011 about 20% of all tissue biopsies in Bangladesh were made at this clinic at which specimens are assessed from patients from all parts of the country. As there is no universal health care, free at the point of service, payment for the investigation would normally be paid by the family. Specimens are either from biopsies carried out at the clinic following abnormal results on imaging or, less frequently, from surgical biopsy conducted elsewhere. All biopsy samples reviewed between 1 January 2008 and 30 October 2011, were extracted and urinary tract specimens identified. Where two or more samples were reported for the same individual on the same day one was retained at random where the result was the same (benign or malignant). Where they differed and one was malignant, that one was retained. A questionnaire developed by MGM had been administered to all patients at the time of enrolment (but before results were available). This questionnaire included information on domicile in the last 10 years, use of drinking water from tube wells and detailed smoking information. Patients with urinary tract specimens (kidney, ureter, bladder, urethra) aged 18 years or greater who reported living in a village for the previous 10 years and drinking water from hand-pumped tube wells were retained for the analysis. In a case-referent design, cases and referents were defined by the histological diagnosis of tissue taken from the ureter, bladder or urethra. Cases were patients diagnosed as TCC: referents were those with a non-malignant (benign) diagnosis.

### 2.1. Arsenic Concentrations in Well Water

The systematic sampling of 3534 wells by the BGS in 1998–1999 had resulted in a mean of eight samples (range 1–16) for each of 443 thanas in 61 of the 64 Districts into which Bangladesh was divided at that time: three Districts in the remote Chittagong hill tracts were excluded. Exposure to arsenic in drinking water was estimated for each subject as the mean arsenic concentration (with “none detected” set at 0.5 µg/L, as in the BGS data) in wells sampled by the BGS for the thana in which the patient lived at the time of biopsy: where the address as extracted did not indicate the thana, the clinical record was reviewed by MGM and assigned to the correct or closest thana, blind to level of arsenic contamination. Arsenic concentrations were categorized into 6 strata: <10 µg/L (less that World Health Organization guideline), 10 < 50 µg/L (less that the Bangladesh guideline), 50 < 100 µg/L, 100 < 200 µg/L, 200 < 300 µg/L and ≥300 µg/L.

### 2.2. Confounding

Information on smoking was collected from the patient or near relative at the time the tissue specimen was collected or delivered for analysis. This was classified as never smoker, past smoker and current smoker. The total years smoking and number of cigarettes was also recorded. Because of some uncertainty whether all ex-smokers had been recorded in the database, all males (or their next of kin) recorded as non-smokers were re-contacted for the present analysis to ascertain whether they had ever smoked and where a history of past smoking was obtained, the data were corrected. In the analyses reported here smoking is included only as “ever” or “never” smoked. No information was collected on exposure to smokeless tobacco.

### 2.3. Statistical Methods

The risk of TCC compared to benign lesions of the ureter, bladder or urethra was examined by concentration of arsenic in drinking water in an unmatched logistic regression analysis, allowing for clustering within thana, adjusting for age and stratifying by sex and smoking habit. The analysis used the STATA multilevel modeling routine (gllamm). Differences in mean arsenic exposure were examined by histological diagnosis and site of tissue biopsy. In a supplementary analysis, the association of arsenic concentration to diagnosis was examined within the referent series, estimating the risk of diagnosis of chronic cystitis (CC) compared to other benign lesions, again using an unmatched logistic regression analysis adjusted for clustering within thana and for confounding by age, sex and smoking. Finally, a case referent analysis was carried out using CC alone as the referent group to explore factors differentiating TCC from CC.

## 3. Results and Discussion

### 3.1. Results

**Figure 1:**
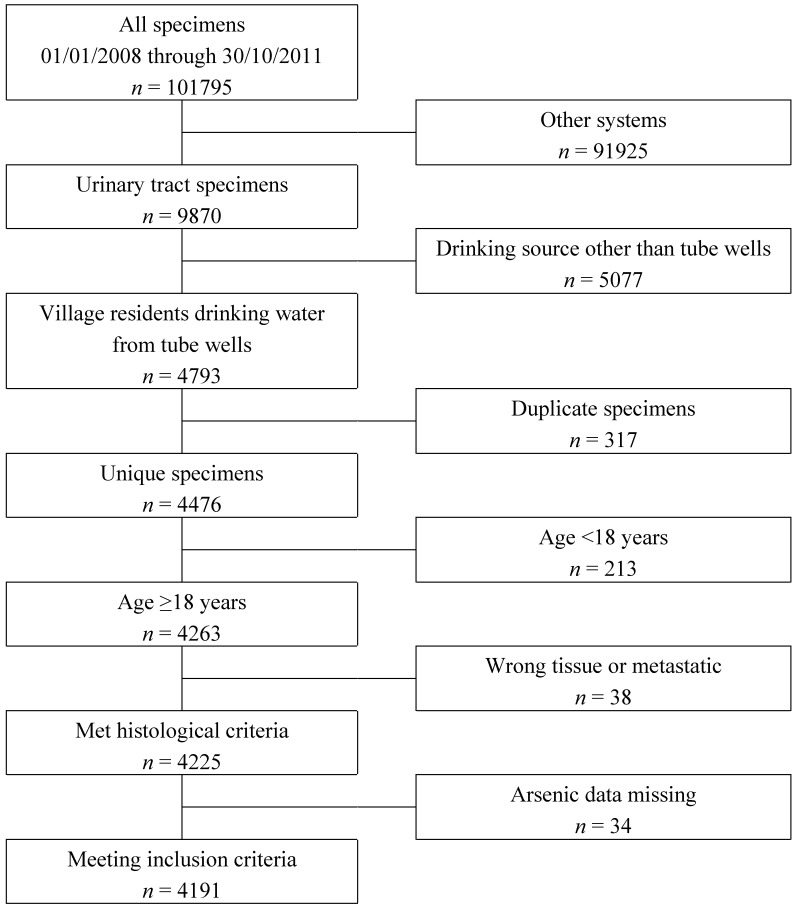
Data available for the analysis of urinary tract lesions.

Of the 9870 patients with a urinary tract biopsy, 4793 had lived in a village for the past 10 years and reported using tube-well water for drinking. After eliminating duplicate samples for the same subject, this number was reduced to 4476. Of these 2368 were specimens from the urinary bladder, 1700 renal tissue, 259 from the ureter and 149 from the urethra. There were 213 samples from patients aged <18, 38 where the sample was from an organ or metastatic tumour outside the urinary tract and 34 were excluded because there was no data on arsenic concentration or because the median date of well installation was unknown. In all, 285 were excluded as meeting one or more of these criteria, leaving 4191 with urinary tract specimens and information on arsenic concentration (Figure 1).

Subjects were drawn from all 61 districts with exposure data from the BGS and were living in 360 of the 433 thanas. Among the patients’ samples retained there were 1522 renal, 236 ureter, 2293 bladder and 140 urethra. The distribution by cell type and sex is shown in Table 1. More detail on the benign diagnoses by site is given as supplementary Table S1: almost all diagnoses of cystitis were recorded as chronic. Rather more than half the samples (54.7%) were from the urinary bladder. Among these 2293 samples, 60.3% were given the histological diagnosis of TCC. These comprised 90.0% (1383/1536) of the TCC diagnoses from throughout the urinary tract.

The distribution of the 2524 patients with TCC or benign diagnoses excluding the kidney (for which results had been published [1]) is shown by sex, age and smoking in Table S2. The majority (74.0%; 1875/2524) of samples were from men. In both men and women those with TCC were older than those with benign bladder lesions and were more likely to have smoked cigarettes (although only 24 women reported smoking). The distribution of cases (TCC) and referents (all benign diagnoses) by estimated arsenic concentration is shown in Table 2.

Among these 2524 patients only 953 (37.8%) lived in thana where the mean arsenic concentration was <50 µg/L (and only 17.6% below the WHO guideline of 10 µg/L). For women there was a somewhat positive trend (Table 3) between arsenic exposure and a diagnosis of TCC but for men, while there was an increased risk for those with exposure estimated to be 10 ≤ 50 µg/L compared to those with exposures <10 µg/L, at higher concentrations, particularly in smokers, no increased risk was apparent (Table 3).

**Table 1:** Histological diagnosis by tissue within the urinary tract by sex.

Histological Diagnoses	Kidney			Ureter			Bladder			Urethra			All		
	Male	Female	Both	Male	Female	Both	Male	Female	Both	Male	Female	Both	Male	Female	Both
Benign															
*n*	281	222	503	101	72	173	513	280	793	48	64	112	943	638	1581
%	27.5	44.3	33.0	68.7	80.9	73.3	29.0	53.5	34.6	72.7	86.5	80.0	31.4	53.7	37.7
Transitional cell carcinoma															
*n*	61	29	90	40	14	54	1171	212	1383	2	7	9	1274	262	1536
%	6.0	5.8	5.9	27.2	15.7	22.9	66.2	40.5	60.3	3.0	9.5	6.4	42.4	22.1	36.6
Renal cell carcinoma															
*n*	657	239	896	-	-	-	-	-	-	-	-	-	657	239	896
%	64.3	47.7	58.9	-	-	-	-	-	-	-	-	-	21.9	20.1	21.4
Other malignant															
*n*	22	11	33	6	3	9	86	31	117	16	3	19	130	48	178
%	2.2	2.2	2.2	4.1	3.4	3.8	4.9	5.9	5.1	24.2	4.1	13.6	4.3	4.0	4.2
All															
*n*	1021	501	1522	147	89	236	1770	523	2293	66	74	140	3004	1187	4191
%	100.0	100.0	100.0	100.0	100.0	100.0	100.0	100.0	100.0	100.0	100.0	100.0	100.0	100.0	100.0

**Table 2:** Distribution of TCC and benign diagnoses (ureter, bladder, urethra) by arsenic concentration (µg/L) by sex.

Arsenic Concentration µg/L	Men				Women				Both				All	
	TCC		Benign		TCC		Benign		TCC		Benign			
	*n*	%	*n*	%	*n*	%	*n*	%	*n*	%	*n*	%	*n*	%
≤10	211	17.4	126	19.0	27	11.6	80	19.2	238	16.5	206	19.1	444	17.6
10 < 50	278	22.9	101	15.3	41	17.6	89	21.4	319	22.1	190	17.6	509	20.2
50 < 100	177	14.6	91	13.7	27	11.6	54	13.0	204	14.1	145	13.5	349	13.8
100 < 200	219	18.1	154	23.3	59	25.3	90	21.6	278	19.2	244	22.6	522	20.7
200 < 300	207	17.1	95	14.4	44	18.9	48	11.5	251	17.4	143	13.3	394	15.6
300 or more	121	10.0	95	14.4	35	15.0	55	13.2	156	10.8	150	13.9	306	12.1
Total	1213	100.0	662	100.0	233	100.0	416	100.0	1446	100.0	1078	100.0	2524	100.0
X^2^ (df = 5)	28.2				13.5				23.7				-	
*tabl*	*p* < 0.001				*p* < 0.019				*p* < 0.001				-	

**Table 3:** Risk of TCC compared to benign lesions (ureter, bladder, urethra) logistic regression allowing for clustering.

	Men						Women		Both	
	Smoking		Non-Smoking		Both					
	OR	95% CI	OR	95% CI	OR	95% CI	OR	95% CI	OR	95% CI
Arsenic µg/L										
<10	1	-	1	-	1	-	1	-	1	-
10 < 50	1.70	1.01–2.86	1.67	0.97–2.89	1.68	1.14–2.48	1.41	0.71–2.81	1.52	1.08–2.14
50 < 100	1.06	0.61–1.83	1.10	0.61–1.97	1.05	0.68–1.60	1.41	0.65–3.06	1.07	0.73–1.57
100 < 200	0.68	0.40–1.13	0.99	0.58–1.69	0.80	0.54–1.12	1.90	0.95–3.81	0.99	0.69–1.41
200 < 300	0.90	0.49–1.63	2.19	1.20–4.01	1.29	0.81–2.07	3.38	1.54–7.44	1.63	1.08–2.46
300 or more	0.65	0.34–1.25	0.65	0.34–1.26	0.66	0.39–1.11	2.06	0.89–4.78	0.89	0.55–1.43
Smoking										
Never	–	–	–	–	1	–	–	–	1	–
Ever	–	–	–	–	2.47	2.00–3.05	–	–	2.71	2.20–3.33
Sex										
Female	–	–	–	–	–	–	–	–	1	–
Male	–	–	–	–	–	–	–	–	1.46	1.16–1.85
Age (years)	1.03	1.02–1.04	1.04	1.03–1.05	1.04	1.03–1.04	1.05	1.04–1.06	1.04	1.03–1.05

Table 4 shows the mean arsenic concentration in well water in the thana of residence by histological diagnosis throughout the urinary tract. For the kidney the previously reported relationship [1] between arsenic concentration and both TCC and RCC is again apparent, subjects with TCC (mean concentration 158.6 µg/L) and RCC (146.7 µg/L) having mean arsenic concentrations markedly higher than those with benign lesions (80.1 µg/L) (TCC (F = 34.5, *p* < 0.001): RCC (F = 81.3, *p* < 0.001)). Among those with lesions of the ureter, patients with TCC again had higher mean arsenic concentrations (177.9 µg/L) than those with CC and other benign lesions (89.2 µg/L) (F = 29.6, *p* < 0001) but this pattern was not present for lesions of the bladder or urethra. For the bladder those with TCC had significantly lower estimates of arsenic concentration (130.6 µg/L) than those with benign lesions (152.1 µg/L) (F = 13.4, *p* < 0.001). No significant difference in arsenic concentration was seen for those with TCC or benign lesions of the urethra (F = 1.2, *p* = 0.3).

**Table 4:** Mean estimated arsenic concentration (µg/L) in wells of thana of residence by histological diagnoses and site.

Histological Diagnosis	Origin of Tissue Sample														
	Kidney			Ureter			Bladder			Urethra			All		
	Mean	SD	*n*	Mean	SD	*n*	Mean	SD	*n*	Mean	SD	*n*	Mean	SD	*n*
Benign															
Chronic cystitis	-	-	-	98.3	89.3	125	153.8	137.3	726	81.5	98.1	33	143.3	132.2	884
Other	81.0	111.0	503	65.4	102.1	48	133.4	147.5	67	70.4	102.1	79	83.8	114.5	697
Transitional cell cancer (TCC)	158.6	137.4	90	177.9	133.8	54	130.6	127.2	1383	35.9	46.7	9	133.4	128.3	1536
Renal cell cancer (RCC)	146.7	140.6	896	-	-	-	-	-	-	-	-	-	146.7	140.6	896
Other malignant	86.6	116.0	33	149.4	179.7	9	99.6	110.8	117	83.3	105.7	19	97.9	115.1	178
All	124.4	134.5	1522	111.8	114.1	236	136.5	131.0	2293	72.6	98.8	140	128.6	131.0	4191

Within those with benign lesions of the ureter, bladder and urethra, patients with lesions classified as CC had, at each site, higher arsenic concentration than those with other benign diagnoses. This difference was highly significant (F = 25.5, *p* < 0.001) when the data for the three sites was considered together, with a mean of 143.3 µg/L in those with CC and 83.8 µg/L for those with other benign diagnoses. When only bladder lesions were considered, and where the proportion of benign lesions other than CC was <10%, the estimated arsenic concentration (153.8 µg/L) for those with the diagnosis of CC was not significantly higher than those with other benign diagnoses (133.3 µg/L) (F = 1.34, *p* = 0.2).

Two additional analyses were carried out to examine further the relation between CC and arsenic exposure. First, among those with non-malignant lesions, the data were examined to determine whether arsenic exposure was related to CC, in a multilevel model allowing for clustering within thana and for age, stratifying by sex and smoking. The numbers for this analysis were small (only one male smoker with arsenic concentration ≥300 µg/L had a diagnosis other than TCC or CC) and the three highest concentration stata were collapsed to allow estimates of risk. For both men and women, those living in a thana with arsenic concentration ≥100 µg/L had more than twice the risk of a diagnosis of CC (rather than “other benign”) compared to those with exposure <10 µg/L. The risk was four-fold in men who smoked (Table 5).

The second analysis, undertaken on the premise that arsenic in drinking water could cause both TCC and CC, considered which factors were related to the TCC diagnosis in a case referent analysis with only CC as the referent, again using a multilevel model allowing for clustering within thana. In this analysis (Table S3) age and, for men, smoking were strong determinants of whether the diagnosis recorded was TCC rather than CC, For women arsenic concentration did not add significantly to the model (F = 5.76 with 5 d.f., *p* = 0.3). For men, very high arsenic concentrations (≥300 µg/L) were less likely to be associated with a diagnosis of TCC than CC (OR = 0.54, 95% CI 0.32–0.91), particularly in male smokers (OR = 0.49, 95% Cl= 0.25–0.95).

**Table 5:** Relation of arsenic concentration to histological cystitis: patients with CC (*n* = 884) compared to those with benign lesions other than CC (*n* = 194).

		Women		Men					
				Never Smoker		Ever Smoker		All	
Arsenic concentration	*n*	OR *	95% CI	OR *	95% CI	OR *	95% CI	OR **	95% CI
<10 µg/L	206	1	-	1	-	1	-	1	-
<50 µg/L	190	0.95	0.38–2.41	1.28	0.47–3.52	2.27	0.64–8.06	1.72	0.75–4.00
50 < 100 µg/L	145	1.24	0.41–3.81	0.72	0.27–1.88	1.69	0.47–6.10	0.96	0.42–2.18
≥100 µg/L	537	2.46	1.01–5.97	1.73	0.73–4.10	4.01	1.47–10.91	2.44	1.20–4.94

* Adjusted for age and clustering within thana; ** Adjusted for age, ever smoker and clustering within thana.

### 3.2. Discussion

The analysis reported here is the first to examine data on the relation between arsenic in drinking water and extra-renal transition cell cancer in Bangladesh. The method adopted, comparing estimated exposure concentrations in those with malignant and benign lesions diagnosed from a single clinic, had provided coherent results in examining lung [16] and renal [1] cancer but the present analysis failed to support the large body of data from which the conclusion has been drawn that arsenic in drinking water causes transition cell cancer, particularly of the bladder [2,3]. In this study no clear dose response was seen. This failure to demonstrate the previously reported relationship might have arisen by chance or because there really is no effect (that is, conclusions previously drawn were erroneous). Alternatively, the absence of effect might be due to some bias within the study design. Potential bias and effects of the inevitable misclassification of exposure using estimates from thana of residence have been discussed in detail in a previous publication [1] but one possible source of bias specific to the present analysis would be if an important number of biopsies read as CC were taken from patients previously treated for TCC and for whom the new biopsy was taken simply to confirm that they were still free of malignant disease. To investigate this we took a 10% random sample of cases with CC of the bladder and one of us (MGM) contacted the patient or family to determine whether the patient had ever been diagnosed with bladder cancer: none had. Having excluded this potential source of bias, the most likely explanation is that bias arose from “over-matching”. This occurs, in a case-referent study, when a condition that is itself caused by the exposure is chosen as the referent group. The data presented here suggest that high arsenic concentrations in drinking water cause symptomatic CC requiring biopsy for diagnosis. The apparent “protective” effect of very high arsenic, associated with a histological diagnosis of CC rather than TCC in male smokers, may indicate that this combination of exposures is particularly likely to result in urinary tract symptoms of inflammation well before any longer latency malignancy. If we accept that both TCC and CC are associated with (or caused by) arsenic in drinking water, this raises the question of whether these are two independent outcomes (in the same way that pleural plaques and mesothelioma are both independently caused by asbestos exposure) or whether CC is, or can be, a precursor of TCC. Chronic inflammation is a risk factor for a number of cancers (for example, inflammatory bowel disease for colon cancer [17]) and has been postulated as a factor for TCC [18]. In the present study those diagnosed with CC (mean age 52.1 years) were some 8 years younger than those with TCC (mean age 60.3 years) but exposed to at least the same concentrations of arsenic. It would be prudent to suppose that those diagnosed with CC in areas of high arsenic concentration are at risk of progressing to malignancy. Given the relative ease of both monitoring for and treating bladder cancer, follow-up of patients with symptomatic CC in high arsenic areas may allow for early diagnosis and treatment. Moreover, with good record keeping, it would provide valuable information on rates of transition to malignancy.

## 4. Conclusions

If we accept that arsenic in drinking water does cause TCC, as concluded by IARC, these data suggest that arsenic also leads to CC, with the resultant “over-matching” masking in this study the expected relation between arsenic concentration and risk of TCC. Given that a diagnosis of CC is based on histology consistent with chronic inflammation and the postulated role of chronic inflammation in TCC, we hypothesize that CC is on the causal pathway for TCC and that those diagnosed with CC in high arsenic areas should be considered at high risk for subsequent malignancy.

## Supplementary Files

Supplementary File 1
