# Supplementary material for: Arsenic in Drinking Water, Transition Cell Cancer and Chronic Cystitis in Rural Bangladesh
Source: Int J Environ Res Public Health. 2015 Oct 28;12(11):13739–49. doi: 10.3390/ijerph121113739 (PMC4661611; doi:10.3390/ijerph121113739)
Supplement: Supplementary File 1 [file ijerph-12-13739-s001.pdf]

Arsenic in Drinking Water, Transition Cell Cancer and Chronic Cystitis in Rural Bangladesh

Table S1. Diagnosis of benign lesion by site of tissue.

Diagnosis	Kidney		Ureter		Bladder		Urethra	
	n	**%**	***n***	**%**	***n***	**%**	***n***	**%**
Cystis	–	–	125	72.3	726	91.6	33	29.4
Tuberculosis	18	3.5	10	5.8	30	3.8	0	–
Cyst	186	37	1	0.6	0	–	2	1.8
Benign tumor	65	12.9	10	5.8	31	3.9	6	5.4
Hydronephritis	111	22.1	–	–	–	–	–	–
Chronic nonspecific urethritis	–	–	25	14.5	_	_	19	17
Chronic pylonephritis	63	12.5	–	–	–	–	–	–
Urethral caruncle	–	–	–	–	–	–	42	37.5
Other	60	11.9	2	1.2	6	0.8	10	8.9
Total	503	100.0	173	100	793	100.0	112	100

Table S2. Distribution of TCC and benign diagnoses (ureter, bladder, urethra) by age and smoking by sex.

Age (years)	TCC						Benign						All					
	Men		Women		Both		Men		Women		Both		Men		Women		Both	
	n	%	*n*	%	*n*	%	*n*	%	*n*	%	*n*	%	*n*	%	*n*	%	*n*	%
18 < 45	110	9.1	36	15.5	146	10.1	187	28.2	197	47.4	384	35.6	297	15.8	233	35.9	530	21.0
45 < 55	208	17.1	57	24.6	265	18.3	137	20.7	83	20.0	220	20.4	345	18.4	140	21.6	485	19.2
55 < 65	351	28.9	75	32.2	426	29.5	135	20.4	72	17.3	207	19.2	486	25.9	147	22.7	633	25.1
≥65	554	44.8	65	27.9	609	42.1	203	30.7	64	15.4	267	24.8	747	39.8	129	19.9	876	34.7
Total	1213	100.0	233	100.0	1446	100.0	662	100.0	416	100.0	1078	100.0	1875	100.0	649	100.0	2524	100.0
Cigarette smoker																		
Never	341	28.1	209	89.7	550	38.0	334	50.5	416	100.0	750	69.6	675	36.0	625	96.3	1300	51.5
Ever	872	71.9	24	10.3	896	62.0	328	49.5	0	-	328	30.4	1200	64.0	24	3.7	1224	48.5
Total	1213	100.0	233	100.0	1446	100.0	662	100.0	416	100.0	1078	100.0	1875	100.0	649	100.0	2524	100.0

X^2 (TCC v benign); Age (df=4) men 136.1 $p < 0.001$, women 70.1 $p < 0.001$, both 272.5 $p < 0.001$; Smoking (df=1) men 92.8 $p < 0.001$, women 44.5 p < 0.001, both 2449 $p < 0.001$.

Table S3. Relation of arsenic concentration to transitional cell cancer (TCC): patients with histological diagnosis of TCC or cystis of the ureter, bladder or urethra (adjusted for clustering within thana and site of lesion).

Classification	Women		Men					
			Never Smokers		Ever Smokers		All	
Arsenic concentration (μg/L)	OR	95% CI	OR	95% CI	OR	95% CI	OR	95% CI
<10	1	–	1	–	1	–	1	–
10 < 50	1.55	0.73–3.27	1.34	0.75 –2.40	1.39	0.79 - 2.43	1.39	0.92–2.10
50 < 100	1.43	0.62–3.28	1.17	0.62–2.20	0.91	0.50 - 1.66	1.03	0.65–1.62
100 < 200	1.82	0.87–3.80	1.02	0.58–1.80	0.62	0.35 - 1.07	0.78	0.52–1.19
200 < 300	2.64	1.13–6.14	1.65	0.89–3.06	0.68	0.37 - 1.24	1.00	0.63–1.57
≥300	1.69	0.69–4.11	0.55	0.25–1.08	0.49	0.25 - 0.96	0.54	0.32–0.91
Age (years)								
18 < 45	1	–	1	–	1	–	1	–
45 < 55	3.75	2.13–6.59	2.64	1.48–4.70	2.12	1.31–3.45	2.35	1.62–3.39
55 < 65	5.67	3.21–9.64	3.93	2.26–6.83	3.40	2.14–5.41	3.67	2.58–5.22
≥65	4.97	2.83–8.74	3.68	2.20–6.13	3.46	2.25–5.31	3.60	2.59–4.97
Ever smoked								
No	–	–	–	–	–	–	1	-
Yes	–	–	–	–	–	–	2.16	1.73–2.71

© 2015 by the authors; licensee MDPI, Basel, Switzerland. This article is an open access article distributed under the terms and conditions of the Creative Commons Attribution license (http://creativecommons.org/licenses/by/4.0/).
